# Knowledge, Attitudes, and Practices of Community Pharmacy Professionals on Poultry Antibiotic Dispensing, Use, and Bacterial Antimicrobial Resistance in Zambia: Implications on Antibiotic Stewardship and WHO AWaRe Classification of Antibiotics

**DOI:** 10.3390/antibiotics11091210

**Published:** 2022-09-07

**Authors:** Steward Mudenda, Moses Mukosha, Brian Godman, Joseph Fadare, Sydney Malama, Musso Munyeme, Christabel Nang’andu Hikaambo, Aubrey Chichonyi Kalungia, Audrey Hamachila, Henson Kainga, Flavien Nsoni Bumbangi, Victor Daka, Ruth Lindizyani Mfune, Geoffrey Mainda, Webrod Mufwambi, Prudence Mpundu, Maisa Kasanga, Shereen Ahmed Mohammed Saad, John Bwalya Muma

**Affiliations:** 1 Department of Pharmacy, School of Health Sciences, University of Zambia, Lusaka P.O. Box 50110, Zambia; 2 Department of Disease Control, School of Veterinary Medicine, University of Zambia, Lusaka P.O. Box 32379, Zambia; 3 Africa Center of Excellence for Infectious Diseases of Humans and Animals, University of Zambia, Lusaka P.O. Box 32379, Zambia; 4 Department of Public Health Pharmacy and Management, School of Pharmacy, Sefako Makgatho Health Sciences University, Pretoria 0208, South Africa; 5 Centre of Medical and Bio-Allied Health Sciences Research, Ajman University, Ajman 346, United Arab Emirates; 6 Department of Pharmacoepidemiology, Strathclyde Institute of Pharmacy and Biomedical Science (SIPBS), University of Strathclyde, Glasgow G4 0RE, UK; 7 Department of Pharmacology and Therapeutics, Ekiti State University College of Medicine, Ado-Ekiti 362103, Nigeria; 8 Department of Biological Sciences, School of Natural Sciences, University of Zambia, Lusaka P.O. Box 32379, Zambia; 9 Department of Veterinary Epidemiology and Public Health, Faculty of Veterinary Medicine, University of Agriculture and Natural Resources, Lilongwe P.O. Box 219, Malawi; 10 School of Medicine, Eden University, Lusaka P.O. Box 37727, Zambia; 11 Michael Chilufya Sata School of Medicine, Copperbelt University, Ndola P.O. Box 21692, Zambia; 12 Department of Veterinary Services, Central Veterinary Research Institute, Ministry of Fisheries and Livestock, Lusaka P.O. Box 50060, Zambia; 13 Department of Environmental and Occupational Health, Levy Mwanawasa Medical University, School of Health Sciences, Lusaka P.O. Box 33991, Zambia; 14 College of Public Health, Zhengzhou University, Zhengzhou 450001, China; 15 College of Veterinary Science, University of Bahr El-Ghazal, Wau P.O. Box 10739, South Sudan

**Keywords:** antibiotic use, antibiotic resistance, antimicrobial resistance, antimicrobial stewardship, attitudes, AWaRe classification, knowledge, pharmacists, poultry, practices, surveillance

## Abstract

Globally, the inappropriate dispensing and use of antibiotics in animals has contributed to the development of bacterial antimicrobial resistance (AMR). In Zambia, there is insufficient information among community pharmacy professionals on antibiotic use (ABU) and AMR in food-producing animals. This study assessed community pharmacy professionals’ knowledge, attitudes, and practices regarding poultry antibiotic dispensing, use, and bacterial AMR in the Lusaka district of Zambia. A cross-sectional study was conducted among 178 community pharmacy professionals between February and April 2022 using a semi-structured questionnaire. Data were analyzed using Stata version 17. Of the total participants (*n* = 178), 51.1% (*n* = 91) were pharmacists. The most dispensed antibiotic was oxytetracycline, a Watch antibiotic, mainly without prescriptions. Good knowledge of ABU and AMR was associated with work experience for more than one year (*p* = 0.016), while good practices were associated with male gender (*p* = 0.039) and work experience of more than one year (*p* = 0.011). The study found moderate knowledge, positive attitudes, and moderate practices of pharmacy professionals on poultry ABU and AMR. There was high dispensing of poultry antibiotics without prescriptions, which calls for strict implementation of antimicrobial stewardship and surveillance programs in poultry production in Zambia to reduce AMR.

## 1. Introduction

In recent years, there has been an increase in the use of antibiotics in poultry farming for improved egg production and growth promotion, as well as the prevention and treatment of infections across countries arising from increased intensification of farming [1,2,3,4,5]. This increase in antibiotic use (ABU) in poultry production has been due to an increased global demand for chicken meat and eggs in recent years [6], with over 119 billion tonnes of chicken meat produced in 2019 and 121 billion tonnes being produced in 2022, with production expected to rise [7,8]. The global chicken meat sales were USD 192.3 billion in 2019, up 3.8% over 2018 [7]. Unfortunately, inappropriate ABU in this sector has contributed to the development of bacterial antimicrobial resistance (AMR) [9,10,11]. Bacterial AMR affects the globe negatively and now accounts for appreciable challenges in treating human infections, food security, and the healthcare system [12,13,14,15], leading to increased morbidity and mortality across countries [16,17]. Overall, there were an estimated 4.95 million deaths globally in 2019 associated with bacterial AMR, the greatest in sub-Saharan Africa, which, if unchecked, will reduce the income of countries by up to USD 3.4 trillion by 2030, equivalent to 3.8% of annual gross domestic product [17,18,19].

Poultry has been reported to be a source of proteins and income for many people across the globe, leading to increased demand for poultry products [2,3,20]. Due to this increased demand, most poultry farmers have resorted to using antibiotics regularly for increased egg production, growth promotion, disease prevention and treatment without observing the withdrawal period when chicken meat and eggs are regarded as ready for human consumption [2,16,21,22,23]. The Food and Agriculture Organization (FAO) and the World Health Organization (WHO) described this behavior as a lack of responsible use of antibiotics [24]. Furthermore, FAO and WHO have appealed to livestock farmers to desist from this practice to reduce the development and spread of AMR [24]. The indiscriminate use of antibiotics in poultry production may result in the accumulation of these drugs in poultry products [25]. This continuous use of antibiotics in poultry has caused an increase in antibiotic-resistant microorganisms [12,26,27] that may be transmitted to humans from poultry through the food chain [28,29,30,31,32,33].

Overall, the access to antibiotics among poultry farmers, often without prescriptions, has worsened the problem of bacterial AMR [34,35]. Antibiotics should be dispensed by trained professionals, including pharmacists, pharmacy technologists, and veterinary professionals, as they are the major custodians of antibiotics [36,37]. During dispensing of antibiotics to farmers, professionals are required to provide adequate information on their appropriate use in poultry. However, most pharmacy and veterinary professionals do not advise farmers on the appropriate use of antibiotics for their flocks, including necessary withdrawal periods [38,39,40]. Withdrawal periods are seen as a necessity for antibiotics as this prevents consumers from unnecessary intake of antibiotics via the food chain. Moreover, most antibiotics are typically obtained from agrovets that are not operated by animal and human health professionals [39,41]. This practice is a concern as it deprives farmers of important information regarding the appropriate use of antibiotics in the poultry sector [42]. Consequently, this calls for continuous training on antimicrobial stewardship (AMS) and surveillance of bacterial AMR among pharmacy and veterinary professionals and farmers, given the existing high levels of resistance to commonly used antibiotics in the human and animal sectors in Zambia [43].

Globally, the use of antibiotics in both layer and broiler production systems has been reported [6,44,45]. Most antibiotics used in poultry production are also used in humans and share the same drug classification, mechanisms of action, and side-effects [46]. Some antibiotics currently used include tetracycline, amoxicillin, erythromycin, gentamicin, doxycycline, sulfadimidine, and sulfamethoxazole/ trimethoprim and are usually accessed without prescriptions [47]. Unfortunately, many poultry microorganisms have developed resistance to these antibiotics [3,45,48]. Similar information has been reported among African countries [3,29,48,49,50] and this generally calls for more prudent use of antibiotics.

AMS and surveillance programs may help reduce bacterial AMR by promoting the rational use of antibiotics [51,52,53,54] as well as facilitating training on ABU and AMR among farmers, animal health professionals, and healthcare workers [15,52,55]. Community pharmacists are key members of any AMS teams because they are in constant contact with poultry farmers in communities and are well placed to contribute positively to reducing the burden of bacterial AMR [54,56,57,58]. They can also continuously monitor ABU and AMR among poultry farmers as well as help develop appropriate activities to improve the future use of antibiotics. In addition, community pharmacy professionals can collaborate with animal health providers to curb bacterial AMR in poultry through improved monitoring and other activities [52,59]. This includes monitoring ABU using the World Health Organization (WHO) “Access”, “Watch”, and “Reserve” (AWaRe) classification of antibiotics protocol [60]. The AWaRe classification tool is crucial in monitoring rational ABU, optimizing antibiotic use, developing AMS programs, and curbing bacterial AMR [60,61]. Consequently, pharmacy professionals must utilize this tool and use it to promote the rational use of antibiotics in the poultry sector.

Understanding the knowledge, attitudes, and practices (KAP) of professionals who prescribe and dispense antibiotics used in poultry is crucial in promoting rational ABU and curbing AMR [14,15,62]. Additionally, the KAP of poultry farmers must also be assessed as they are heavily involved in purchasing and administering antibiotics to their birds [63]. Insufficient knowledge and awareness of poultry ABU and bacterial AMR have been reported to contribute to the inappropriate prescribing and dispensing of antibiotics [64,65]. Consequently, understanding the KAP of pharmacy professionals is a positive strategy for developing pertinent programmes for curbing AMR in the future.

Currently, Zambia, a country in sub-Saharan Africa, faces the challenge of AMR in human and animal medicine [41,43,66,67,68]. The increased use of antibiotics in poultry in sub-Saharan Africa has contributed to the problem of AMR [65]. Some studies have reported AMR isolates from poultry that have been linked to the indiscriminate use of antibiotics among Zambian poultry farmers [48,69]. To tackle AMR, the Zambian government developed a Multi-sectoral National Action Plan using a “One Health” approach with strategies to combat AMR in animals, humans, and the environment [70]. However, despite the burden of AMR in poultry, there currently appears to be no published or documented information on the KAP of community pharmacists and pharmacy technologists on poultry ABU and AMR. Moreover, these professionals are among the first point of contact offering healthcare services for humans and animals. The products they dispense include veterinary medicines including antibiotics used in poultry. Consequently, this study was conducted to assess the KAP of community pharmacy professionals on the dispensing of poultry antibiotics, ABU and AMR in Lusaka, Zambia. This is key to guiding policymakers on the strategies for early detection, prevention, and management of AMR.

## 2. Results

Overall, 178 participants participated in the study, of which 91 (51.1%) were pharmacists and 87 (48.9%) were pharmacy technologists. The largest proportion, 79 (44.4%) of participants were between 26 and 33 years old, and 91 (51.1%) had more than five years of working experience. More than half, 103 (57.9%) of the participants were males, 95 (53.4%) were married, and 136 (76.4%) resided in urban areas (Table 1).

**Figure 1:**
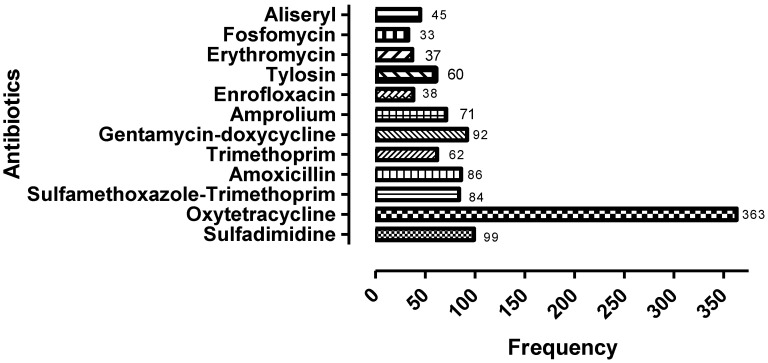
Distribution of commonly dispensed antibiotics by pharmacists and pharmacy technologists.

The most dispensed antibiotics in retail pharmacy outlets for poultry use were oxytetracycline (33.9%) followed by sulfadimidine (9.3%), gentamicin/doxycycline (gentadox) (8.5%), and amoxicillin (8.0%) (Figure 1).

Most (96.6%) of the participants were aware of AMR, knew about the use of antibiotics in poultry (54.5%), discouraged the addition of antibiotics to chicken feed (73.6%), and encouraged farmers not to stop administration of antibiotics until the course was complete (86.5%). Most (73%) participants dispensed antibiotics without prescriptions (Table 2).

### 2.1. Respondents’ Knowledge, Attitudes, and Practices of ABU and AMR

Table 3 shows the cross-tabulation of participants’ characteristics and mean knowledge, attitude, and practice scores. Overall, the mean (SD) knowledge, attitude, and practice scores were 64.7 (18.0), 81.3 (11.4), and 56.6 (16.7), respectively. Consequently, participants had moderate knowledge, positive attitudes, and moderate practices. The scores for knowledge and practice were not significantly different between pharmacists and pharmacy technologists. However, the scores for attitude were significantly (*p* = 0.049) higher for pharmacy technologists, 83.0 (12.1), than for pharmacists, 79.7 (10.6). Furthermore, there was a significant difference in knowledge scores between male pharmacy technologists and pharmacists, with the former recording higher scores (69.3 vs. 61.9). In addition, pharmacy technologists aged between 26 and 33 years had higher scores for attitude than pharmacists of a similar age group (83.2 vs. 78.2), staying in urban areas (83.5 vs. 79.3) and with less than one-year working experience (90.6 vs. 72.9).

### 2.2. Factors Associated with Mean Knowledge, Attitude, and Practice Scores of ABU and AMR

The results (Table 4) demonstrated that the specialty (pharmacists or pharmacy technologists) was not independently associated with knowledge, attitude, and practice of ABU and AMR. On the other hand, the scores for knowledge (regression coefficient (β), −0.13; 95% confidence interval (CI), −0.23 to −0.02) and practice (β, −0.36; 95% CI: −0.12 to −0.02) were independently lower for respondents with less than one year of working experience than the respondents with 1–5 years of working experience. Similarly, males had higher scores for practice than females (β = 0.05; 95% CI: 0.01 to 0.10).

Overall, there was a significant but moderate positive correlation between knowledge and attitude (r = 0.23, *p* = 0.002), knowledge and practice (r = 0.38, *p* < 0.001) and attitude and practice (r = 0.26, *p* < 0.001) (Table 5).

## 3. Discussion

To the best of our knowledge, this is the first study in Zambia to assess community pharmacy professionals’ knowledge, attitudes, and practices (KAP) on ABU and AMR for antibiotics used in poultry production. Overall, there was moderate knowledge, positive attitudes, and moderate practices regarding poultry ABU and AMR. The most dispensed antibiotics used in poultry production were oxytetracycline, followed by gentamicin+doxycycline (gentadox) and amoxicillin. A brief review of KAP studies in different countries presented various findings in relation to our study.

The overall knowledge and practices concerning ABU and AMR among our participants were moderate, contrasting with a study in the Kingdom of Bhutan in which most veterinarians and veterinarian assistants had good knowledge of AMR building on the Bhutan National Plan of AMR [71]. A multi-country study in Ethiopia, India, Nigeria, the Philippines, Sierra Leone, and Vietnam also reported a high awareness of AMR among human and animal healthcare professionals [72]. Nevertheless, this did not translate into a reduction in prescribing and dispensing antibiotics [72]. This contrasts with findings from Bangladesh, where most drug sellers were unaware of AMR [73], Fiji, where most para-veterinarians were not aware of AMR [62], and Ethiopia, where farm owners and their employees were generally unaware of key activities surrounding ABU [63]. Alongside this, in Nigeria, most veterinary students were also unaware of AMR and its contributing factors [74]. Similarly, in Grenada, a study among poultry farmers and employees found a low awareness of AMR [75], and in Tanzania, there was generally low public awareness of ABU and AMR [76]. This low awareness of poultry ABU and AMR may suggest the need for enhanced educational programs among all stakeholders [77].

The low knowledge of ABU and AMR reported in these studies could be due to a lack of participation in AMR activities, including awareness weeks, continuous professional development, and not attending refresher courses. Our study found that the pharmacy professionals generally had positive attitudes regarding ABU and AMR. This is important as positive attitudes can influence the practice and help improve the future use of antibiotics in poultry. Similarly, favorable attitudes were reported in the Kingdom of Bhutan among veterinarians and para-veterinarians [71]. In Bangladesh, drug and feed sellers had less positive attitudes and greater inappropriate practices regarding AMU and AMR [73].

The moderate practices reported in our study can be improved by adding more information regarding veterinary medicine into the pharmacy curriculum and promoting greater collaboration (“One Health” approach) between the two professions [78]. In addition, the gaps regarding KAP reported in our and similar studies [71,73] require improvements in the training curriculum, routine instigation of continued professional development and education, sensitization and campaign programs, as well as other educational activities on ABU and AMR in the poultry sector to improve future ABU [52,79,80,81,82].

Of concern is that the indiscriminate use of antibiotics in the poultry sector in Zambia was similar to other studies [83,84,85], which needs to be changed. The most dispensed antibiotics in poultry production were oxytetracycline, gentamicin+doxycycline (gentadox), amoxicillin, and sulfamethoxazole-trimethoprim, which is similar to studies conducted in Kenya and Uganda, where tetracyclines, especially oxytetracycline, were the most used antibiotics by poultry producers [63,86,87]. In Ethiopia, tetracyclines were the most used antibiotics in poultry farms, followed by penicillin [63]. Furthermore, in a study that included five African countries, tetracyclines were among the most dispensed and used antibiotics in poultry, followed by macrolides and aminoglycosides [42]. The increased use of oxytetracycline in poultry could be due to its activity against several bacteria and the cost/effectiveness ratio which attracts most farmers to purchase it [88]. This is a growing concern with high resistance rates to tetracyclines reported across countries [1,10,26,89,90,91,92]. However, different from some studies conducted in Bangladesh and France in which the most dispensed antibiotics were fluoroquinolones followed by tetracyclines [73,85]. In addition, in China, the most dispensed and misused antibiotics were amoxicillin followed by norfloxacin [93], and in Tanzania, the commonly dispensed class of antibiotics for poultry use were fluoroquinolones, followed by sulfonamides [94]. These antibiotics for poultry use are typically obtained without prescriptions [2,21,22,93] and often the choice of antibiotic is based on information obtained from social networks [38,42]. Consequently, poultry farmers miss out on expert information [86], which can potentially lead to inappropriate and excessive use of antibiotics contributing to AMR [93,95]. According to the WHO AWaRe classification of antibiotics, the highly dispensed antibiotics in our study (tetracyclines) and similar studies (tetracyclines and fluoroquinolones) belonged to the “Watch” group of antibiotics [60,61]. This practice is inappropriate since the majority of antibiotics prescribed and dispensed should belong to the “Access” group rather than the “Watch” and “Reserve” groups to reduce resistance potential [96,97,98]. Consequently, there is an urgent need to address this concept as a part of AMS activities in animals and humans.

There are also gaps in the awareness of AMR and the links between ABU and AMR in our study, similar to other studies [67,71]. This suggests a need for pharmacy professionals to work closely with veterinary professionals and related stakeholders in developing and instigating strategies to curb unnecessary ABU to prevent the adverse consequences of AMR [99]. This can build on our findings that most of the participants in our study knew about the uses and withdrawal periods of antibiotics, similar to studies in Bangladesh and Bhutan [71,73]. Despite these gaps, most (94%) of the study participants felt that antibiotics are misused in poultry production, similar to other studies [16,23,44,64,73,100]. As a result, this may create an opportunity for pharmacy and veterinary professionals to collaborate and curb the inappropriate use of antibiotics in the poultry sector to reduce AMR, similar to other countries to optimize animal care [73,99].

Most participants in our study felt that restricting the use of antibiotics in poultry would be beneficial, along with instigating guidelines to improve the prescribing and dispensing of antibiotics. This is because continuous access to antibiotics in poultry production without prescriptions is a global challenge requiring appropriate action and policies [82,91,101]. The instigation of veterinary prescription policies is a potential strategy to control ABU and curb rising AMR in poultry [82], with the need for restricting access to and use of antibiotics in poultry production, including for growth promotion, supported by others [102,103,104,105], with the high use of antibiotics in poultry production without prescriptions [38,42,86,93,106], currently influenced by customer and price preferences [39]. These are among the major factors contributing to the development of AMR [42,86,106]. Up-to-date and robust guidelines can also help promote the appropriate use of antibiotics, along with guidance on the dose, time, duration, and route of administration [71,107]. Guidelines should also promote the use of vaccines in poultry disease prevention and the application of disease preventive measures (biosecurity) rather than using antibiotics [107]. Alternative strategies could also include the use of prebiotics, probiotics, feed enzymes, synbiotics, and phytogenic feed additives which should be promoted in the poultry sector [108,109]. Nevertheless, the prudent use of antibiotics is the best strategy to reduce future AMR [63,110].

On a positive note, most participants in our study encouraged poultry farmers to maintain the withdrawal period and not to sell poultry products when still using antibiotics, which, as mentioned, is important in preventing the passage of resistance to humans [111,112]. The failure of poultry farmers to observe the antibiotic withdrawal periods may result in the accumulation of drug residues in poultry products that can be passed on to humans through the food chain [111,113], which needs to be avoided giving rising AMR rates globally especially in sub-Saharan Africa [17]. A similar situation was seen in Bangladesh, where the majority of poultry drug sellers were aware of the withdrawal period [73] alongside veterinary practitioners in the same country [114]. However, most poultry farmers in Tanzania were not compliant with the withdrawal periods [94], with a similar situation observed in Kenya [2]. This requires educational programs among poultry farmers on the importance of antibiotic withdrawal periods. However, the pharmacy professionals in our study encouraged the poultry farmers to stop administering antibiotics to flocks immediately after the flocks recovered from a disease. A similar practice was reported among drug sellers in Bangladesh [73]. Eventually, a follow-up study in the same country revealed that some farmers stopped administering antibiotics when the birds felt better [64]. This practice is inappropriate because antimicrobial courses must be completed even if the flocks recover from an infection.

Encouragingly as well, our findings indicated that the majority (84%) of pharmacy professionals referred poultry farmers to animal health workers for specialized services, indicating a good collaboration between pharmacy and veterinary professionals to help reduce the inappropriate use of antibiotics among poultry [115]. There is a need for continuous interaction between human and animal disease experts involved in managing animal diseases with a deliberate focus on the “One Health” approach to reduce AMR in humans [99]. This is because veterinarians are essential in diagnosing poultry disease, prevention, and treatment [116]. Moreover, the veterinary experts can help promote the rational use of antibiotics in poultry and curb AMR [117]. Further, collaborations between veterinary professionals and other stakeholders can also be helpful in the surveillance of AMR in poultry [118].

This study also demonstrated that knowledge of ABU and AMR among pharmacy professionals was influenced by years of work experience. Professionals who had worked for one year and above had better knowledge of ABU and AMR. However, a study among veterinarians reported that good knowledge of ABU and AMR was reported among those that read the National Action Plan on antibiotics and AMR [71]. Another study reported that good knowledge of ABU and AMR was influenced by age, level of education, years of experience, and training on ABU and AMR [114]. Our study also demonstrated that the practice of pharmacy professionals was influenced by their years of work experience in which those who had worked for one year and above had better practices concerning ABU and AMR. These findings corroborate with the reports from other studies [64,73]. Based on these findings, the Zambian animal and human healthcare workers should familiarize themselves with the Multi-sectoral National Action Plan on AMR [70]. This can, in turn, improve their knowledge and practices regarding ABU and AMR.

Further, our study found that participants’ knowledge, attitudes, and practices on ABU and AMR were related. Increasing participants’ knowledge through education and training activities on ABU and AMR should improve their attitudes and practices leading to improved use of antibiotics in the future. However, using a cross-sectional study to predict the interventions’ outcomes is impossible. Additionally, our study did not include questions whether pharmacy professionals had asked poultry farmers on previous access to antibiotics for use in their flocks. Nevertheless, this study provides vital information that can be used to help develop context-specific strategies more likely to restrict the prescribing and dispensing of antibiotics for poultry use in the future. As an ongoing study, we recommend future studies that may predict outcomes of introducing educational campaigns and training activities on antibiotic use in poultry and AMR among pharmacy professionals. Furthermore, we recommend the implementation of evidence-based practices that focus on behavior change of all stakeholders (pharmacy professionals, veterinary professionals, and farmers) involved in handling and administration of antibiotics used in poultry. This will be based on strategies that have been successfully introduced in a number of sectors and situations to improve future ABU [18].

## 4. Materials and Methods

### 4.1. Study Design and Site

A cross-sectional study was conducted among community pharmacists and pharmacy technologists in Zambia’s Lusaka province from February 2022 to April 2022. Lusaka district was purposively selected because it had the largest number of poultry farmers at the time of the study [41]. Moreover, this district currently has most of the community pharmacies in Zambia [119,120]. Poultry farmers accessed most of the poultry antibiotics in community pharmacies and agrovets [41].

### 4.2. Study Population and Sample Size Estimation

This study was conducted among community pharmacy professionals (pharmacists and pharmacy technologists) working in the Lusaka district. Community pharmacies and the pharmacy professionals registered with the Zambia Medicines Regulatory Authority (ZAMRA) and the Health Professions Council of Zambia (HPCZ) were eligible for the study. No community pharmacist was excluded due to their age. However, we excluded community pharmacies that did not stock poultry antibiotics at the time of the study. We also excluded veterinarians and agrovets as they do not practice in community pharmacies. In Zambia, every registered community pharmacy is under the responsibility of a pharmacist and assisted by pharmacy technologists. The number of registered community pharmacies under the responsibility of a registered pharmacist in Lusaka was obtained from the ZAMRA website [119]. Thus, a finite population of 370 pharmacy professionals based on 370 registered community pharmacies was obtained and used in sample size determination at a 5% margin of error. The sample size was determined using the Raosoft sample size calculator at a 95% confidence level and 50% response distribution. A 50% response distribution was used since there were no similar published studies in Zambia. A sample size of 189 pharmacy professionals was estimated. The identified participants from each registered pharmacy were selected using a simple random sampling technique. Overall, 178 participants were included in the study.

### 4.3. Data Collection Tool

The data collection tool was adapted from a similar study by Kalam and colleagues [73], reviewed for content and validated by experts from the University of Zambia. The internal consistency was acceptable, with a Cronbach’s alpha value above 0.76 for all the questions on knowledge, attitude, and practice of AMR and ABU. The questionnaire included socio-demographic characteristics of study participants, knowledge, attitude, and practice questions regarding poultry ABU and AMR (Supplementary Material). Based on a previous study [121], good knowledge was determined as scores above 82%; moderate knowledge was scored from 55% to 82% and low knowledge was scored below 55%. Positive attitudes were scores of 63% and above, while negative attitudes were scores below 63%. Good practices were assessed as scores above 58%, moderate practices were from 35% to 58%, and poor practices were below 35%. Actual scores for knowledge, attitude and practice were calculated by adding correct/positive responses (coded as 1 for correct/positive response, and otherwise zero). A pilot study was conducted among 15 pharmacy professionals to validate the questionnaire; later, these findings were excluded from the data analysis. The data were collected by three data collectors using a self-administered questionnaire that lasted between 10 and 20 min. See supplementary material for the attached questionnaire.

### 4.4. Statistical Analysis

Descriptive statistical methods were used to assess socio-demographic characteristics by the mean score for knowledge, attitude, and practice of ABU and AMR. Student *t*-test was used to compare the mean differences across explanatory variables since the knowledge, attitude, and practice scores were normally distributed. The normality test was done graphically using QQ-plots and Shapiro–Wilk test.

Three general linear regression models were fitted with knowledge, attitude, and practice scores as outcomes, respectively. First, a univariable linear regression model was fitted with socio-demographic characteristics. Candidate variables with *p* < 0.20 in univariable analysis were included in the multivariable linear regression model, which included specialty (Pharmacists vs. Pharmacy technologists). Stepwise regression and backward elimination algorithms were used with a liberal *p*-value for exclusion (*p* < 0.15) to fit the multivariable model. Only the specialty was fixed in the multivariable models because it was set as a priori variable.

Studentized residuals and the Cook–Weisberg test for heteroskedasticity were used to assess the goodness of fit of the models. Finally, we checked for linear relationships (using fractional polynomial model comparisons) under model-checking procedures and the variance inflation factors (VIF) to check for multicollinearity. In the final model, none of the factors reached a VIF value of 5; thus, multicollinearity was not a problem in this case. Stata/BE version 17 (Stata Corporation, College Station, Brazos County, TX, USA) was used in all the statistical analyses. Significance was considered when *p* < 0.05.

## 5. Conclusions

This study found that the most dispensed antibiotics for poultry use in Zambia are tetracyclines, sulfonamides, aminoglycosides, and penicillins. The pharmacists and pharmacy technologists had moderate poultry ABU and AMR knowledge and practices. Conversely, their practice demonstrated some gaps that require a multi-sectoral one-health approach in tackling AMR, as well as continuous professional development (CPDs) training on the use of antibiotics in poultry. Further, based on our findings with most young professionals having poor practices, there is a need to strengthen the pharmacy training curriculum on poultry diseases and ABU may improve their practice. Alongside this, there is a need to develop and strengthen strategies that promote the prudent use of antibiotics in poultry. The development of AMS and surveillance programs may be one way forward to address the AMR challenges faced in Zambia and other nations. Community pharmacy professionals should be included in any future AMS programs given their importance in this and other areas, and we will be monitoring this in the future. We will also be following up with veterinarians and agrovet personnel in the future, especially those working in rural areas, as key stakeholders with improving future ABU among poultry.

## Figures and Tables

**Table 1:** Socio-demographic characteristics of study participants.

Variable	Level	Frequency (%)
Age (years)	18–25 26–33 34–41 42–49	22 (12.4) 79 (44.4) 59 (3.2) 18 (10.1)
Sex	Female Male	75 (42.1) 103 (57.9)
Specialty	Pharmacist Pharmacy technologist	91 (51.1) 87 (48.9)
Work experience (years)	<1 1–5 >5	16 (9.0) 71 (39.9) 91 (51.1)
Residence	Rural Urban	42 (23.6) 136 (76.4)

**Table 2:** The proportion of pharmacists and pharmacy technologists who correctly/positively responded to knowledge, attitudes, and practices questions on poultry ABU and AMR.

Variable	Knowledge, Attitude, and Practice Questions	Total *n* = 178 (%)	Pharmacists *n* = 91 (%)	Pharm Techs *n* = 87 (%)	*p*-Value
Knowledge	I know the withdrawal period of antimicrobials	120 (67.4)	65 (71.4)	55 (63.2)	0.245
	I am aware of antimicrobial resistance	172 (96.6)	91 (100)	81 (93.1)	**0.011**
	Antimicrobials can cure all diseases caused by microorganisms in the poultry	97 (54.5)	43 (47.3)	54 (62.1)	**0.047**
	Antibiotics are effective against viral infections	34 (19.1)	14 (15.4)	20 (23.0)	0.197
	All antimicrobials can have the same curative effect in poultry diseases	59 (33.2)	30 (33.0)	29 (33.3)	0.959
	Antimicrobials have some side effects	161 (90.5)	84 (92.3)	77 (88.5)	0.388
	Antimicrobials are required for all flocks; when one bird is sick	125 (70.2)	60 (65.9)	65 (74.7)	0.200
	Antimicrobials residues can be passed to humans from poultry products	153 (86.0)	80 (87.9)	73 (83.9)	0.442
Attitude	Use of antimicrobials in poultry may lead to AMR	170 (95.5)	89 (97.8)	81 (93.1)	0.132
	Missing dose of antimicrobials in poultry can lead to AMR	168 (94.4)	88 (96.7)	80 (92.0)	0.169
	Restriction on antimicrobial usage in poultry can be more beneficial than harmful	161 (90.5)	80 (87.9)	81 (93.1)	0.239
	Antimicrobials should be added with feed to prevent diseases at any time	47 (26.4)	16 (17.6)	31 (35.6)	**0.006**
	Antimicrobials should be stored in a designated place in the shop	171 (96.1)	88 (96.7)	83 (95.4)	0.655
	Antimicrobials should be sold at a less price when about to expire to prevent wastage	99 (55.6)	46 (50.6)	53 (60.9)	0.164
	We need guidelines for dispensing poultry antimicrobials	175 (98.3)	89 (97.8)	86 (98.9)	0.587
	Antimicrobials are misused in poultry production	167 (93.8)	84 (92.3)	83 (95.4)	0.391
Practice	I sell poultry antimicrobials without a prescription	130 (73.0)	71 (78.0)	59 (67.8)	0.125
	I recommend farmers to use antimicrobials as growth promoters	33 (18.54)	14 (15.4)	19 (21.8)	0.268
	I encourage farmers to observe withdrawal periods	139 (78.1)	72 (79.1)	67 (77.0)	0.734
	I ask farmers to increase the dose and frequency when poultry disease persists	33 (18.5)	16 (17.6)	17 (19.5)	0.737
	I recommend farmers to stop using antimicrobials before completing the course when poultry gets improved	24 (13.5)	7 (7.7)	17 (19.5)	**0.021**
	I encourage farmers not to sell poultry products during the use of antimicrobials	143 (80.3)	74 (81.3)	69 (79.3)	0.736
	I inform farmers about the course of antimicrobials	155 (87.1)	77 (84.6)	78 (89.7)	0.316
	I refer poultry farmers to veterinary experts for specialist services	149 (83.7)	80 (87.9)	69 (79.3)	0.120

All values are mean percentage scores with standard deviation (SD), *p*-values from students’ Chi-square test/Fisher’s exact test/student *t*-test. Pharm techs-pharmacy technologists, boldface indicates statistical significance at *p* < 0.05.

**Table 3:** Cross-tabulations: Socio-demographic characteristics and mean knowledge, attitude, and practice scores among pharmacists and pharmacy technologists.

Variables	Mean Knowledge Score (SD)			Mean Attitude Score (SD)			Mean Practice Score (SD)		
	Pharmacist	Pharm Tech	*p*-Value	Pharmacist	Pharm Tech	*p*-Value	Pharmacist	Pharm Tech	*p*-Value
Age (years)									
18–25	66.3 (21.3)	50.0 (16.0)	0.054	80.0 (12.1)	82.2 (18.0)	0.736	56.3 (27.2)	52.1 (20.5)	0.686
26–33	65.4 (16.6)	69.6 (17.6)	0.278	78.2 (11.4)	83.2 (9.3)	**0.035**	56.1 (13.3)	57.7 (16.2)	0.640
34–41	63.9 (15.2)	65.2 (23.8)	0.794	81.3 (10.1)	82.1 (13.0)	0.788	57.3 (12.5)	57.6 (15.4)	0.931
42–49	58.8 (16.7)	64.0 (10.4)	0.445	78.8 (8.4)	85.9 (14.1)	0.197	55.0 (20.6)	56.3 (28.3)	0.915
Sex									
Female	68.4 (17.4)	61.0 (19.1)	0.093	79.7 (8.2)	82.6 (12.2)	0.256	55.1 (13.8)	51.2 (17.8)	0.305
Male	61.9 (15.6)	69.3 (19.3)	**0.033**	79.7 (11.8)	83.5 (12.0)	0.106	57.2 (16.6)	62.2 (16.1)	0.128
Work experience (years)									
1–5	68.5 (16.9)	64.9 (17.9)		80.2 (10.8)	81.3 (12.4)	0.707	61.2 (15.1)	56.5 (19.0)	0.274
>5	64.0 (15.5)	67.4 (20.9)	0.391	81.0 (10.2)	84.1 (11.5)	0.171	56.8 (14.6)	57.9 (17.2)	0.724
<1	54.2 (16.3)	46.9 (15.7)	0.379	72.9 (10.4)	90.6 (12.0)	**0.013**	46.9 (6.3)	43.8 (15.5)	0.706
Residence									
Rural	61.8 (19.4)	61.4 (18.8)	0.954	81.3 (13.0)	81.8 (11.0)	0.889	58.3 (21.0)	56.8 (17.7)	0.795
Urban	64.7 (15.8)	66.7 (19.8)	0.526	79.3 (10.0)	83.5 (12.5)	**0.029**	56.0 (14.1)	56.7 (16.0)	0.785
Overall group scores	64.1 (16.5)	65.2 (19.6)	0.690	79.7 (10.6)	83.0 (12.1)	**0.049**	56.5 (15.6)	56.7 (17.8)	0.906
Overall scores	64.7 (18.0)			81.3 (11.4)			56.6 (16.7)		

SD—standard deviation, boldface indicates statistical significance at *p* < 0.05.

**Table 4:** Linear regression of factors associated with mean knowledge, attitude, and practice scores of ABU and AMR.

Variable	Knowledge		Attitude		Practice	
	β, 95% CI	*p*-Value	β, 95% CI	*p*-Value	β, 95% CI	*p*-Value
Specialty						
Pharmacist	Ref		Ref		Ref	
Pharmtech	−0.01 (−0.06, 0.05)	0.942	0.03 (−0.01, 0.07)	0.056	−0.01 (−0.05, 0.05)	0.989
Age (years)			-		-	
18–25	Ref					
26–33	0.06 (−0.03, 0.16)	0.173				
34–41	0.02 (−0.09, 0.13)	0.756				
42–49	−0.02 (−0.14, 0.11)	0.782				
Sex	-		-			
Female					Ref	
Male					0.05 (0.01, 0.10)	**0.039**
Work experience (years)						
1–5	Ref		Ref		Ref	
>5	0.01 (−0.06, 0.07)	0.821	0.02 (−0.01, 0.06)	0.253	−0.01 (−0.06, 0.05)	0.807
<1	−0.13 (−0.23, −0.02)	**0.016**	−0.02 (−0.09, 0.04)	0.467	−0.12 (−0.21, −0.02)	**0.011**
Residence			-		-	
Rural	Ref					
Urban	0.05 (−0.02, 0.11)	0.148				

NB: β-regression coefficient, 95% CI—95% confidence intervals, -variable dropped from the model; the models were fitted using scores of knowledge, attitude and practice, respectively, as continuous variables. In all the models, the specialty was retained as a priori variable; boldface indicates statistical significance at *p* < 0.05.

**Table 5:** The correlation between knowledge, attitude, and practice of ABU and AMR.

Variables	Correlation Coefficient	*p*-Value
Knowledge-attitude	0.23	0.002
Knowledge-practice	0.38	<0.001
Attitude-practice	0.26	<0.001

## Data Availability

The data supporting the reported results can be made available on request from the corresponding author.

## Supplementary Materials

The following supporting information can be downloaded at: https://www.mdpi.com/article/10.3390/antibiotics11091210/s1, Questionnaire.
