# Supplementary material for: Knowledge, Attitudes, and Practices of Community Pharmacy Professionals on Poultry Antibiotic Dispensing, Use, and Bacterial Antimicrobial Resistance in Zambia: Implications on Antibiotic Stewardship and WHO AWaRe Classification of Antibiotics
Source: Antibiotics (Basel). 2022 Sep 7;11(9):1210. doi: 10.3390/antibiotics11091210 (PMC9495135; doi:10.3390/antibiotics11091210)
Supplement: Supplementary file 1 [file antibiotics-11-01210-s001.zip › antibiotics-1842339-supplementary.pdf]

antibiotics

MDPI

Supplementary Materials

Knowledge, Attitudes, and Practices of Community Pharmacy Professionals on Poultry Antibiotic Dispensing, Use, and Bacterial Antimicrobial Resistance in Zambia: Implications on Antibiotic Stewardship and WHO AWaRe Classification of Antibiotics

Steward Mudenda [1,2,3,*], Moses Mukosha [1], Brian Godman [4,5,6], Joseph Fadare [7], Sydney Malama [8], Musso Munyeme [2], Christabel Nang'andu Hikaambo [1], Aubrey Chichonyi Kalungia [1], Audrey Hamachila [1], Henson Kainga [2,9], Flavien Nsoni Bumbangi [2,10], Victor Daka [3,11], Ruth Lindizyani Mfune [11], Geoffrey Mainda [12], Webrod Mufwambi [1], Prudence Mpundu [13], Maisa Kasanga [14], Shereen Ahmed Mohammed Saad [3,15] and John Bwalya Muma [2]

[1] Department of Pharmacy, School of Health Sciences, University of Zambia, Lusaka P.O. Box 50110, Zambia
[2] Department of Disease Control, School of Veterinary Medicine, University of Zambia, Lusaka P.O. Box 32379, Zambia
[3] Africa Center of Excellence for Infectious Diseases of Humans and Animals, University of Zambia, Lusaka P.O. Box 32379, Zambia
[4] Department of Public Health Pharmacy and Management, School of Pharmacy, Sefako Makgatho Health Sciences University, Pretoria 0208, South Africa
[5] Centre of Medical and Bio-allied Health Sciences Research, Ajman University, Ajman 346, United Arab Emirates
[6] Department of Pharmacoepidemiology, Strathclyde Institute of Pharmacy and Biomedical Science (SIPBS), University of Strathclyde, Glasgow G4 0RE, UK
[7] Department of Pharmacology and Therapeutics, Ekiti State University College of Medicine, Ado-Ekiti **362103**, Nigeria
[8] Department of Biological Sciences, School of Natural Sciences, University of Zambia, Lusaka P.O. Box 32379, Zambia
[9] Department of Veterinary Epidemiology and Public Health, Faculty of Veterinary Medicine, University of Agriculture and Natural Resources, Lilongwe P.O. Box 219, Malawi
[10] School of Medicine, Eden University, Lusaka P.O Box 37727, Zambia
[12] Department of Veterinary Services, Central Veterinary Research Institute, Ministry of Fisheries and Livestock, Lusaka P.O. Box 50060, Zambia
[13] Department of Environmental and Occupational Health, Levy Mwanawasa Medical University, School of Health Sciences, Lusaka P.O. Box 33991, Zambia
[14] Zhengzhou University, College of Public Health, 100 Kexue Avenue, Henan 450001, China
[15] College of Veterinary Science, University of Bahr El- Ghazal, Wau P.O. Box10739, South Sudan
* Correspondence: freshsteward@gmail.com; Tel.: +260-977549974

Questionnaire #: …………………*Instructions: please tick (√) where appropriate*

SECTION A: Sociodemographic characteristics of participants

1. What is your gender? A. male [ ] b. female [ ]
2. How old are you? a.18-25 [ ] b. 26-33 [ ] c.34-41 [ ] d. 42-49 e. Above 50 years [ ]
3. What is your occupation? a. pharmacist [ ] b. pharmacy technologist [ ]
4. Work experience? A. less than 1 year [] b. 1-5 years [] c. Above 5 years []
5. What is your religion? A. Christianity [ ] B. Islam [ ] C. Hinduism [ ] D. Others, specify ……………………………………..
6. What is your marital status? a. Married [ ] b. Single [ ] c. Divorced [ ] d. Bereaved [ ]

7. Which of these describes your current residence? a. Urban [ ] b. Rural [ ] c. Rural urban [ ]

Awareness of poultry antibiotics

1. Do you stock poultry antibiotics?
   i. Yes ii. No
2. If yes, what are the names of poultry antibiotics? [Generic or Trade names can be used]

a. Tetroxy chick formular

b. Tetroxy egg formular

c. Sulfadimidine

d. Sulfamethoxazole-Trimethoprim

e. Trimethoprim

f. Gentamicin-doxycycline

g. Amoxicillin

h. Enrofloxacin

i. Tylosin

j. Amprolium

k. Others (specify)……

Knowledge questions

1. Do you know about withdrawal period of antimicrobials (not eating poultry products during chicken treatment with antimicrobials and specified period after treatment)?
2. Do you know about antimicrobial resistance?
3. Do you know antimicrobials can be treated for all diseases caused by microorganisms?
4. Do you know antibiotics are effective against viral infections?
5. Do you know all antimicrobials can show the same curative effect in poultry diseases?
6. Do you know antimicrobials have some side effects?
7. Do you know antimicrobials are required for all flocks, when one bird is sick?
8. Do you know antimicrobials can be passed to human from poultry products?

Attitude questions

1. I think indiscriminate use of antimicrobials in poultry may lead to AMR
2. I think missing dose of antimicrobials in poultry can lead to AMR
3. I think restriction (under authorisation to prevent use for growth promotion, improved production, and prophylaxis of infections) on antimicrobial usage in poultry can be beneficial than harmful
4. I think antimicrobials should be added with feed to prevent diseases at any time
5. I think antimicrobials should be stored in a designated place of the shop
6. I think antimicrobials should be sold at a less price when about to expire to prevent wastage
7. I think we need guidelines for dispensing poultry antimicrobials
8. I think antimicrobials are misused in poultry production

Practice questions

1. Do you sell poultry antimicrobials without a prescription?
2. Do you recommend farmers to use antimicrobials as growth promoters?
3. Do you encourage farmers to maintain withdrawal period?
4. Do you ask farmers to increase dose and frequency when poultry disease persists?
5. Do you recommend farmers to stop using antimicrobials before completing course when poultry gets improved?
6. Do you encourage farmers not to sell poultry products during the use of antimicrobials?
7. Do you inform farmers about the course of antimicrobials?
8. Do you refer poultry farmers to veterinary experts for specialist services?

Thank for your participation
